# Values and Principles of Integrated Care

**DOI:** 10.5334/ijic.2458

**Published:** 2016-03-30

**Authors:** M. M. N. Minkman

**Affiliations:** University of Tilburg/TIAS, NL

This is the first editorial of the International Journal of Integrated Care to be published by our new open-access publisher, Ubiquity Press. It follows a successful 15-year period supported by Igitur at the University Library Utrecht where IJIC was the first digital journal in their portfolio. However, as integrated care has grown as a service innovation, so the ever increasing numbers of articles being submitted to IJIC means that the ‘incubation’ period of our Journal within Igitur has long since passed and so we now enter into a new venture to support a larger audience of academics, professionals and decision-makers keen to absorb the latest knowledge in this growing field of scientific inquiry.

Entering such an exciting new period for our Journal raises the question about what aspects of integrated care require the generation of new knowledge that is backed up by work of high scientific quality? As we know, numerous questions about integrated care have yet to be answered. For example, previous editorials have pointed to the need for new domains of investigation from understanding how to embed integrated care into the community [1] and how to research the outcomes and costs of integrated care properly [2].

For myself, the phenomenon of integrated care remains attractive (almost mysterious) because it seems that we have a lot more to learn beyond the knowledge of ‘how to do it’ and whether integrated care can achieve the (Triple Aim) results to which it aspires. The pluriformity of integrated care and the belief that ‘no form or size fits all’ (not all clients, nor all professionals, nor all systems) draws us back to a discussion on the core purpose of integrated care. What is the basis of integrated care thinking? What lies underneath it? What is it all about?

In my own work I have studied whether generic elements of integrated care could be defined and what these elements are. These studies showed that these elements could indeed be defined and were formulated as activities that seem relevant in multiple and very different integrated care settings. Examples of 89 found ‘elements’ are for instance ‘systematically assessing the needs of clients’ or ‘stimulating trust among care partners’ [3]. A number of following studies showed that these kinds of (generic) elements are relevant in multiple settings regardless of client group, the range of (health and social) care partners involved, the geographical location or even country context [4]. The recently developed taxonomy and Rainbow model [5] also searches for generic knowledge about (the level and type of) integration, and the work of Project INTEGRATE will seek to develop a similar framework to help guide managers and decision-makers later this year.

What these studies show is that there is continued interest in, the conceptual understanding of integrated care and its subsequent underlying principles. It is interesting to see that the 2002 paper of Kodner and Spreeuwenberg [6] – about the concept, meaning and logic of integrated care – remains in the top ten of the most requested IJIC articles. However, if we accept that integrated care strives to improve quality of care and experiences to clients, then better understanding the values that underpin integrated care from that perspective is important and the definitions that we currently accept may need to be challenged.

Recently, Ferrer and Goodwin set out a list of 16 principles of integrated care drawing on their work with the World Health Organisation and the reflection of expert participants from different country contexts [7] [see Box 1]. Ferrer and Goodwin invited readers to join the debate about whether a set of principles is needed and whether the principles that they suggest are the right ones. Whilst the reactions of fellow healthcare scientists and professionals were positive on the need for a set of values and principles, there was considerable difference in opinion on what should, or should not, be included and/or on how certain key principles should be described Specifically, the principles appeared to many to be lacking the perspective of the service user and community and remained driven by the viewpoint of care professionals or the health system.

These discussions suggest that further research and debate is needed to establish the core values and principles to integrated care and, especially, to ensure the set of principles properly includes the perspectives of clients, civilians and communities enough.

In order to address this, Vilans and IFIC have initiated a Special Interest Group (SIG) with the aim of developing a valuable, valid and workable set or principles for person-centered and integrated care. The SIG will act as a forum to further discuss the principles and values of integrated care and also to co-create a comprehensive set of principles that deepen our understanding of the values underpinning integrated care. The launch of the SIG on Principles of Integrated Care will be held at the IFIC Conference 2016 in Barcelona which is scheduled for Wednesday May 25^th^ May at 7.30 AM and an on-line forum will be announced shortly. If you are interested in joining this SIG, please feel invited to participate. To get involved, please send an expression of interest by email to n.zonneveld@vilans.nl. Your contribution will be highly valued!

**Prof. Dr. M.M.N. Minkman**

Head of Research & Innovation, Vilans, National Center of Excellence in Long-term Care.

Distinguished Professor, University of Tilburg/TIAS, Innovation of organization and governance of long term integrated care

1. Comprehensive – a commitment to universal health coverage to ensure care is comprehensive and tailored to the evolving health needs and aspirations of people and populations
2. Equitable – care that is accessible and available to all
3. Sustainable – care that is both efficient, effective and contributes to sustainable development
4. Co-ordinated – care that is integrated around people’s needs and effectively coordinated across different providers and settings
5. Continuous – continuity of care and services that are provided across the life course
6. Holistic – a focus physical, socio-economic, mental, and emotional wellness
7. Preventative – tackles the social determinants of ill-health through intra- and inter-sectoral action that promote public health and health promotion
8. Empowering – supports people to manage and take responsibility for their own health
9. Goal oriented – in how people make health care decisions, assess outcomes and measure success
10. Respectful – to people’s dignity, social circumstances and cultural sensitivities
11. Collaborative – care that supports relationship-building, team-based working and collaborative practice across primary, secondary, tertiary care and other sectors
12. Co-produced – through active partnerships with people and communities at an individual, organisational and policy-level
13. Endowed with rights and responsibilities – that all citizens should expect, exercise and respect
14. Governed through shared accountability – between care providers for quality of care and health outcomes to local people
15. Evidence-informed – such that policies and strategies are guided by the best available evidence and supported over time through the assessment of measurable objectives for improving quality and outcomes
16. Led by whole-systems thinking

## Competing Interests

The author declares that they have no competing interests.
